# Updates in rare and not-so-rare complications of acromegaly: focus on respiratory function and quality of life in acromegaly

**DOI:** 10.12688/f1000research.22683.1

**Published:** 2020-07-29

**Authors:** Fabienne Langlois, Gabriela M. Suarez, Maria Fleseriu

**Affiliations:** 1 Centre Hospitalier Universitaire de Sherbrooke, 3001 12e Avenue Nord, Sherbrooke, QC, J1H 5N4, Canada; 2 Pituitary Center, Departments of Medicine and Neurological Surgery, Oregon Health & Science University, 3303 South Bond Avenue, CH8N, Portland, OR, 97239, USA

**Keywords:** Acromegaly, growth hormone excess, respiratory complication, sleep apnea, quality of life, patients reported outcome

## Abstract

Acromegaly is a complex disease with excessive growth hormone and insulin-like growth factor 1 (IGF-1) causing multisystem effects, particularly cardiovascular, respiratory, and metabolic. Psychological concerns and poor quality of life (QoL) are also major disease consequences. This review is intended for clinicians and focuses on the latest developments related to respiratory and QoL effects of long-term growth hormone excess. Along with biochemical disease control, patient treatment satisfaction and outcomes have become major treatment objectives; current knowledge and tools to evaluate and manage this aspect of the disease are described. Sleep apnea syndrome and other derangements of lung function and apparatus, from pathophysiology to treatment, and evaluation tools and determinants of QoL in patients with acromegaly are discussed.

## Introduction

Acromegaly is a disorder almost exclusively caused by a pituitary growth hormone (GH)-secreting adenoma. The high circulating levels of GH and resulting insulin-like growth factor 1 (IGF-1) have pleiotropic effects on many organs ^1^. Ultimately, acromegaly causes an insidious syndrome that severely impacts patient morbidity and quality of life (QoL). Cardiovascular and respiratory complications are frequent comorbidities and are the main causes of mortality among patients ^2, 3^. Cancer risk is also increased ^4^ and recently cancer has been shown to surpass cardiovascular causes as a main cause of death compared with the general population ^5, 6^. However, with advances in treatment and thus improved biochemical control, mortality has diminished over the past decade ^7^. See Table 1 on multisystem acromegaly complications ^2, 8– 12^.

**Table 1.** Multisystem acromegaly complications.

System
Cardiovascular
- Cardiomyopathy characterized by biventricular hypertrophy or fibrosis or both - Hyperkinetic syndrome - Diastolic or systolic dysfunction or both - Arterial hypertension - Valvulopathies - Arrhythmias - Vascular endothelial dysfunction
Musculoskeletal
- Enlargement/elongation of vertebral bodies - Disrupted trabecular bone architecture and vertebral compression fractures - Thoracic spine kyphoscoliosis - Overgrowth of mandible, malocclusion, and increased teeth spacing - Myopathy, including airway muscle fatigue
Metabolic
- Insulin resistance and diabetes mellitus - Dyslipidemia: hypertriglyceridemia and low high-density lipoprotein cholesterol - Hyperandrogenemia with a polycystic ovary syndrome-like effect - Hypertrichosis
Respiratory
- Upper airway narrowing, including tracheal stenosis and tortuosity - Macroglossia - Tracheo-bronchomegaly - Thickening of true and false vocal cords - Increased lung volume - Possible decreased lung compliance - Small airway obstruction - Obstructive and central sleep apnea
Psychological
- Emotional lability - Social withdrawal - Distorted body image - Diminished quality of life
Gastrointestinal
- Increased colonic polyps - Dolichocolon

Cardiovascular complications and cancer have been extensively reviewed as complications of acromegaly ^13^. The focus here is on the latest developments related to respiratory and QoL effects.

## Respiratory complications

### Background

Patients with acromegaly have been reported to have a 1.85 times higher risk of developing respiratory diseases versus the general population ^14^. One of the most common complications of acromegaly is obstructive sleep apnea (OSA); the risk is approximately 10-fold in acromegaly compared with the general population ^15– 17^. OSA can exacerbate cardiovascular dysfunction and is an important contributor to impaired QoL and eventually mortality ^18^. Lesser-known facets of acromegaly-induced respiratory complications affect pulmonary function, oximetry, and exercise capacity ^13, 19^. Excess GH, on the surface, would seem to be beneficial to physical performance; however, it can also have deleterious effects and contribute to significant fatigue and inability to sustain similar workloads compared with controls ^19, 20^. Overall, the mortality rates from respiratory causes are three times higher when compared with that of the general population ^21– 23^ and contribute to up to one fifth of acromegaly-related deaths ^24, 25^.

### Pathophysiology

Mechanisms by which acromegaly is associated with respiratory complications are not clearly understood. However, there are several proposed mechanisms by which GH can alter lung anatomy; the main hypotheses are as follows:

1. Upper respiratory airways. High GH/IGF-1 can lead to deformities of facial bones, hypertrophy of the pharyngeal and laryngeal cartilages, and soft tissue thickening and macroglossia, which ultimately can lead to inspiratory collapse of the hypopharynx, especially during sleep ^2, 14, 26– 28^. Also, generalized tissue edema, related to high sodium and volume reabsorption by the kidney, which contributes to upper airway narrowing, may be observed in patients with acromegaly ^29, 30^. Ultimately, external compression can occur because of goiter or accumulation of fat and contribute to tracheal deviation, tortuosity, and sometimes stenosis ^31^.

2. Lung volumes. GH stimulates lung growth by increasing the number or size of alveoli (or both) ^14, 32, 33^. Data on histopathology of human lungs in patients with acromegaly are lacking, and clinical data from pulmonary function tests sometimes render conflicting results. In general, increased total lung volumes are measured by total lung capacity, vital capacity, and residual volumes, all of which can be 110 to 160% of a predicted value ^26, 34– 39^. This is related to the direct effect of GH/IGF-1 inducing alveoli hyperplasia, alveoli hypertrophy, or an increase in alveoli number or a combination of these ^26, 34^. Increased lung mass has also been objectively measured by computed tomography (CT) densitovolumetry; active acromegaly compared with controls revealed a 25% mass increase: 885 g versus 696 g ( *P* = 0.017) ^37^.

3. Thoracic musculature. GH and IGF-1 exert proliferative but also degenerative effects on smooth muscle, inducing respiratory muscle dystrophia. Myopathy is evident on pathology as muscle fiber degeneration and abnormal collagen are observed ^40, 41^. These findings translate to enhanced muscle fatigue, as demonstrated in a murine model with effects on sternohyodal muscle ^42^.

4. Thoracic bone changes. The axial skeleton is affected in up to 60% of patients, and changes include thickening of soft and cartilaginous tissues, rib cage deformity, formation of osteophytes, and abnormal trabecular bone microarchitecture leading to vertebral fractures and kyphoscoliosis ^38^.

5. Diffusing capacity. Diffusing capacity of the lung for carbon monoxide (DLCO) can also be altered. Some studies reported a lower DLCO ^27^, most showed neutral diffusing capacity ^26, 27, 34, 35, 38, 43^, and some found an increased DLCO ^44, 45^. Higher lung volumes could lead to an increase in DLCO; therefore, diffusing capacity corrected for alveolar volume is likely to be normal in patients with acromegaly ^46^.

6. Lung compliance. Lung elasticity may also change due to excess GH/IGF-1. Decreased lung recoil might be explained by elevated peripheral rib cage resistance caused by hypertrophic muscles and rib cage or caused by obesity ^26^ but could also be related to a higher alveoli size ^43^. Some series did not demonstrate a change (ref 34) in lung elasticity and one (ref 43) showed that patients with acromegaly had higher lung distensibility, which was partially reversible after treatment ^34, 43^. Various components of lung compliance may be differently affected in the disease and may explain the heterogeneity of results.

7. Obstructive disease. Small airway obstruction is evident on the basis of a reduced maximum expiratory flow after 75% of forced vital capacity had been exhaled (FEF75). Moreover, more than half of patients had clinically important reductions (<80% of predicted value) in FEF75 ^38, 47, 48^. Up to 60% of air trapping causing ventilation perfusion mismatches was reported ^38^. This small airway obstruction might be due to increased thickness or tortuosity of the bronchial wall, increased lung volumes, or vascular congestion or a combination of these ^38^.

8. Bronchiectasis was also noted on high-resolution CT but might not be more prevalent than what is observed in healthy subjects ^49^.

### Diagnosis and management

***Sleep apnea in patients with acromegaly.*** In different series, sleep apnea affects up to 80% of patients and represents a 10-fold increased risk compared with the general population ^11, 13, 50^. A main symptom of OSA is daytime sleepiness caused by repeated micro-arousal due to hypoxemic events during sleep ^50^. Acromegaly is predominantly associated with OSA, caused by upper airway obstruction ^2, 18, 51, 52^. However, central sleep apnea also occurs, as about 30% of patients with acromegaly have a central sleep apnea component ^53– 56^. Pathogenesis remains controversial but might be directly GH-mediated or related to associated cardiomyopathy ^56, 57^. Excess GH is associated with central inhibition of the breathing center and may lower the ventilatory response of the respiratory center to carbon dioxide, resulting in a temporary cessation of the respiratory center and respiratory efforts ^56^.

Other factors, notably obesity, concomitant hypothyroidism, and treatment of hypogonadism, also play a role in OSA ^13^. Hypogonadism in male patients with acromegaly and OSA is frequent. Hormonal replacement should be undertaken with caution, especially in cases of severe untreated OSA since there is some evidence that testosterone might deteriorate OSA ^58^.

If sleep apnea is left untreated, it can lead to fatigue, daytime sleepiness, hypogonadism, arterial and pulmonary hypertension, ischemic heart disease, right ventricular failure, cerebrovascular accidents, life-threatening arrhythmias, poor QoL, and cognitive dysfunction ^14, 18, 50, 52, 59, 60^. Usual predictors such as age, male sex, neck circumference, and body mass index (BMI) are factors associated with the onset and severity of OSA, but finger circumference is an additional predictor specific to acromegaly ^11, 18, 61– 64^. Use of the Epworth Sleepiness Scale or the STOP-Bang (snoring, tiredness, observed apnea, high blood pressure, BMI, age, neck circumference, and male sex) questionnaire can help identify patients, in the general population, but more studies are needed in patients with acromegaly, and polysomnography remains key for diagnosis ^18, 50, 65^.

It is still a matter of debate whether OSA severity is associated with disease activity or disease duration or both ^66, 67^. Some reversibility of OSA may be observed once biochemical control is achieved, but a recent meta-analysis confirms that the prevalence of OSA in patients with controlled acromegaly is similar to that in patients with uncontrolled acromegaly ^68^. Nonetheless, acromegaly patients who underwent treatment have been shown to have improvement in both central and peripheral component of sleep-breathing control, especially by reducing soft tissue swelling and macroglossia ^14, 18, 54, 61, 69^. However, changes in bones and cartilaginous structures affecting upper airway anatomy may be irreversible ^13^, and even worsening or *de novo* OSA, partly related to weight gain after cure, may occur ^16, 18^.

In general, prospective studies suggest that more than 50% of patients with controlled acromegaly continue to have persistent OSA ^13, 14, 55^. Most improvement is attained in the first year after treatment ^55^. Pituitary surgery has been reported to improve sleep apnea and decrease the frequency of apneic and hypopneic episodes by 50 to 60% in a surgically cured cohort ^39, 62^. Medical treatment also leads to improvement in apnea–hypopnea index (AHI), as indices of oxygen desaturation, sleep quality, and subjective sleepiness improved after 6 months of octreotide treatment in approximately half of cases ^70^. Intriguingly, AHI changes in patients do not always correlate with GH or IGF-1 levels, and other factors such as age, sex, and smoking also prevail ^71^. Interestingly, one factor influencing AHI is weight, as OSA did not improve and may occur *de novo* in patients who gained weight after treatment ^72^.

Overall, evaluating patients with acromegaly for OSA at diagnosis is mandatory ^73^. A diagnostic procedure such as polysomnography or home sleep apnea testing should be ordered if clinical suspicion exists. Screening of every patient could be done since the pre-test probability is very high. Follow-up by periodic re-evaluation of OSA is warranted at least 1 year after successful treatment ^74^. Attention to weight gain and physiological hormonal replacement in the post-treatment period may help improve OSA ^13^.

***Pulmonary function abnormalities.*** No definite management concerning respiratory insufficiency exists. Biochemical remission does not seem to improve lung function parameters in most patients ^39^. Pulmonary function tests (spirometry and diffusing capacity) may be ordered in a patient with unexplained shortness of breath or decreased exercise capacity, but cardiovascular dysfunction may also be a cause. No specific treatment has been proposed and tested in this population, but either inhalotherapy or other treatment modalities may be individualized according to findings.

## Quality of life

**Figure 1.**
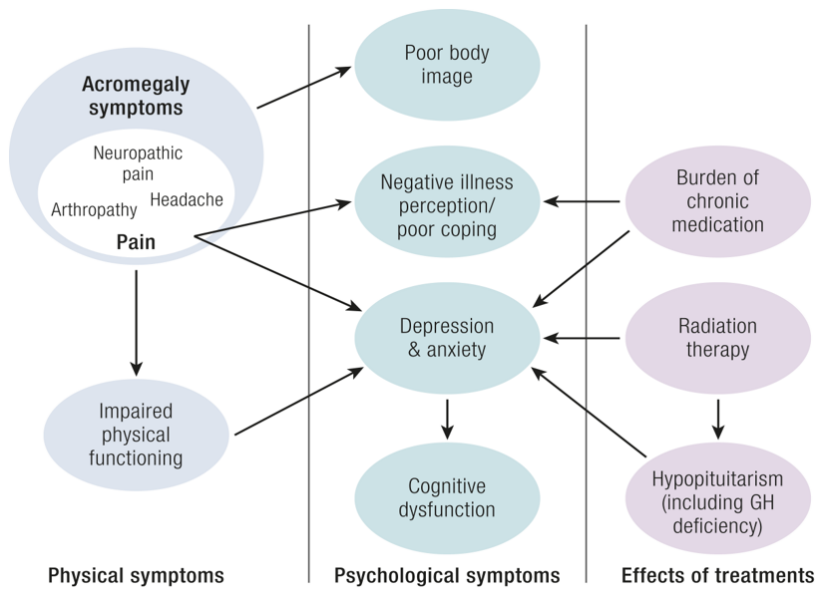
Factors affecting quality of life in patients with acromegaly. [© 2019 Illustration Presentation ENDOCRINE SOCIETY]. GH, growth hormone. The figure was reproduced from Gadelha MR *et al.,* Systemic Complications of Acromegaly and the Impact of the Current Treatment Landscape: An Update, Endocrine Reviews, 2019, Volume 40, Issue 1, Pages 268–332 ^13^ by permission of the Endocrine Society.

Acromegaly is a chronic disease with multiple physical and psychological complications that carry a significant disease burden. Most patients have joint pain, headaches, low energy and libido, and morphological changes such as craniofacial deformation. These changes may not be completely reversible after treatment and can negatively affect QoL ^75^. The comorbidities and active symptomatology also negatively affect patient productivity and entail high costs related to sick leave, unemployment, or disability ^76, 77^. Other factors, such as cognitive dysfunction and restless leg syndrome, are more prevalent in patients with acromegaly and are associated with poor QoL ^78, 79^. Moreover, diagnosis delay and acceptance of the disease could be challenging for patients ^80, 81^. Figure 1 (Gadelha *et al*.) summarizes the factors affecting QoL in acromegaly ^13^.

QoL is defined as the patient’s perception of his or her physical and mental health, cognitive function, anxiety, depression, and subjective feelings of energy levels. QoL is considered an important endpoint in the evaluation of patients with acromegaly. It can be measured with questionnaires designed for use in the general population or aimed at specific disease dimension conditions, such as disease-generated or -specific questionnaires like the Acromegaly Quality of Life (AcroQoL) questionnaire ^75, 82^. Table 2 presents brief details of the following questionnaires: the Symptom Questionnaire ^83, 84^, Signs and Symptoms Score ^85, 86^, the Patient-Assessed Acromegaly Symptom Questionnaire ^85, 86^, Short-Form Health Survey (SF-36) ^87– 89^, the Multidimensional Fatigue Inventory (MFI-20) ^90, 91^, the Nottingham Health Profile ^92, 93^, Health-Related Quality of Life ^12, 94^, Psychological General Well-Being ^82, 95^, the Hospital Anxiety and Depression Scale ^96, 97^, the AcroQoL Questionnaire ^75, 82, 96, 98^, and the Acromegaly Treatment Satisfaction Questionnaire (Acro-TSQ) ^99, 100^.

**Table 2.** Questionnaires to evaluate quality of life in acromegaly.

Questionnaires to evaluate quality of life in acromegaly	Description
Symptom Questionnaire	The Symptom Questionnaire is a 92-item questionnaire assessing four scales: anxiety, depression, somatic symptoms, and anger/hostility. Subjects indicate the frequency of symptoms in the past week on a scale from absent to very often. Higher scores indicate greater symptom severity. This questionnaire may be more sensitive in discriminating between distress levels than other tests ^83, 84^.
Signs and Symptoms Score (SSS)	The SSS questionnaire is designed specifically to measure acromegaly-related signs and symptoms such as headache, perspiration, joint pain, fatigue, and soft tissue swelling. Each of those five questions is evaluated by a score from 0 (absent) to 8 (severe) for a maximum of 40 indicative, very severe clinical manifestations ^85, 86^.
Patient-Assessed Acromegaly Symptom Questionnaire (PASQ)	The PASQ is a disease-specific questionnaire composed of seven questions. The first six questions evaluate physical symptoms (headache, perspiration, joint pain, fatigue, soft tissue swelling, and paresthesia of the extremities) and a seventh question evaluates overall well-being. Each aspect is scored from 0 to 8; a lower score reflects low disease impact and a higher score reflects a more severe disease burden ^85, 86^.
Short-Form Health Survey (SF-36)	The SF-36 is a validated self-administered questionnaire that evaluates physical and psychological symptoms and their impact on daily life. Eight domains are explored: physical functioning, role limitations due to physical functioning, general health perception, pain, vitality, emotional well-being, role limitations due to emotional health, and social functioning. SF-36 consists of 36 questions answered with either “yes/ no” responses or scaled responses ranging from three options (“yes, limited a lot”, “yes, limited a little”, or “no, not limited at all”) to six options (“all of the time”, “most of the time”, “a good bit of the time”, “some of the time”, “a little of the time”, or “none of the time”). Higher scores indicate a better quality of life ^87– 89^.
Multidimensional Fatigue Inventory (MFI-20)	The MFI-20 contains 20 statements to assess fatigue. There are five different dimensions of fatigue and each contains four items. The dimensions are general fatigue, physical fatigue, reduced activity, reduced motivation, and mental fatigue. Items are rated on a scale from 0 (absent) to 5 (extremely). A higher score indicates more fatigue experienced ^90, 91^.
Nottingham Health Profile (NHP)	The NHP is frequently used in patients with pituitary disease to assess general well-being and QoL. This survey consists of 38 “yes/no” questions that are subdivided into six scales assessing impairments: pain (eight items), energy level (three items), sleep (five items), emotional reactions (nine items), social isolation (five items), and disability/functioning (i.e., physical mobility) (eight items). A higher score is associated with more impairment ^92, 93^.
Health-Related Quality of Life (HRQoL)	This is a 15-item, standardized, self-administered HRQoL instrument that can be used both as a profile and as a single-index score measure. The 15 items evaluated are moving, seeing, hearing, breathing, sleeping, eating, speech, eliminating, usual activities, mental function, discomfort and symptoms, depression, distress, vitality, and sexual activity. For each item, the patient chooses one of the five levels that best describes his or her state of health at that moment ^12, 94^.
The Psychological General Well-Being (PGWB)	The PGWB Index is a 22-item questionnaire that evaluates six subscales: anxiety, depressed mood, positive well-being, self-control, general health, and vitality. Each item has six questions, which are scored from 0 to 5. A score of 110 represents a perfect QOL ^82, 95^
Hospital Anxiety and Depression Scale (HADS)	This is a 14-item questionnaire evaluating anxiety and depression. Each item is scored on a 4-point scale. Scores range from 0 to 21 for the anxiety and depression subscale and from 0 to 42 for the total score. Higher scores indicate higher levels of anxiety and depression ^96, 97^.
AcroQoL Questionnaire	The AcroQoL Questionnaire has been used and validated in randomized clinical trials, leading to a better understating of the specific impairments in acromegaly. This questionnaire consists of 22 questions that evaluate physical and psychological dimensions. Answers are recorded on a scale from 1 (always or completely agree) to 5 (never or completely disagree). The maximum score of 110 reflects best possible QoL and is quoted as a percentage (e.g., 100%). The 22 items evaluate physical symptoms such as pain and fatigue and psychological function, such as impact on appearance, performance, and personal relationships ^75, 82, 96, 98^.
Acromegaly Treatment Satisfaction Questionnaire (Acro-TSQ)	This questionnaire is a novel tool to simultaneously evaluate patient satisfaction on disease and treatment with somatostatin receptor ligand. It measures treatment effectiveness, symptom burden, treatment side effects, convenience of treatment, and overall satisfaction over the past 4 weeks ^99, 100^.

A systematic review confirmed that AcroQoL scores are significantly impaired in patients with acromegaly ^101^. Geraedts *et al*. showed that the most significant factors associated with poor QoL were depression and higher BMI ^102^. Other predictors have been inconsistently identified; longer disease duration, painful syndrome (musculoskeletal or headache), diabetes, older age, female gender, having received treatment with radiotherapy, and becoming GH-deficient after treatment ^75, 102, 103^. Successful therapy might improve QoL but may not normalize QoL completely, even after biochemical cure ^75, 104– 107^. Indeed, QoL has been shown to be severely impaired years after successful treatment in patients with acromegaly compared with the general population ^104, 108, 109^.

### Quality of life after treatment

Numerous studies have looked at the impact of different treatment modalities in first- or second-line therapies. Pituitary surgery compared with medical treatment seems superior in improving QoL in patients with acromegaly ^101^ as in other types of pituitary tumor ^110^. A French study found that patients who underwent surgery had a better QoL than if they had received medical treatment only (65 ± 18% versus 54 ± 14%, *P* = 0.009), and investigators concluded that neurosurgery was associated with greater improvement in QoL when compared with medical therapy alone ^111^. Overall, greater GH suppression could be attained with surgery than with somatostatin receptor ligand (SRL) injections ^112^. Indeed, patients with remission after surgery could avoid medication side effects and consequences of suboptimal biochemical control.

Types of medical therapy used rendered heterogeneous results on QoL, see Table 3 ^113– 125^. Some studies with first-line SRL injections in treatment-naïve patients show improved QoL in patients who received long-acting lanreotide ^114, 115^, but the results were controversial ^106, 116, 124, 126^. On the other hand, a multicenter study showed that two thirds of patients controlled on SRL injections reported ongoing incommoding acromegaly symptoms ^127^. In that study, gastrointestinal disturbances were very frequent (73%); this was possibly related to SRL side effects or disease activity itself ^127^. A new questionnaire, Acro-TSQ, was developed to evaluate QoL specifically in patients who received injectable SRLs and provides further insight ^99^.

**Table 3.** Prospective studies on effect of pharmacotherapy on quality of life.

Therapy	Publication	Number of patients	Follow-up, Weeks	Disease status (for majority of patients)	Medical treatment in control group	Scales used	Effect on quality of life
Lanreotide Autogel	Sonino *et al.* (1999) ^113^	10	8	Uncontrolled	Octreotide LAR or bromocriptine	KSQ, CSKSLPP	↑↑
Lanreotide Autogel	Lombardi *et al.* (2009) ^114^	51	52	Uncontrolled	Treatment naïve	NHP	↑↑
Lanreotide Autogel	Caron *et al.* (2016) ^115^	90	48	Uncontrolled	Octreotide LAR	AcroQoL, PASQ	↑↑
Lanreotide Autogel	Schopohl *et al.* (2011) ^116^	37	26–52	Controlled	Octreotide LAR	AcroQoL	↔
Octreotide LAR intensification	Mangupli *et al.* (2014) ^117^	28	24	Uncontrolled	Treatment naïve or Octreotide LAR	AcroQoL	↑↑
Octreotide LAR	Chin *et al.* (2015) ^118^	58	24	Uncontrolled	Treatment naïve	AcroQoL	↑
Octreotide LAR	Ghigo *et al.* (2009) ^119^	57	48	Uncontrolled	Treatment naïve	AcroQoL, SSS	↑↑
Octreotide LAR every 6 weeks	Biermasz *et al.* (2003) ^120^	14	36	Controlled	Octreotide LAR every 4 weeks	NHP	↔
Octreotide LAR or Lanreotide Autogel intensification	Dal *et al.* (2018) ^121^	61	52	Controlled	Octreotide LAR or Lanreotide Autogel	AcroQoL, PASQ	↔
Pasireotide	Bronstein *et al.* (2016) ^122^	119	52	Uncontrolled	Octreotide LAR	AcroQoL	↔
Pegvisomant	Ghigo *et al.* (2009) ^119^	56	48	Uncontrolled	Treatment naïve	AcroQoL, SSS	↑↑
Pegvisomant	Trainer *et al.* (2009) ^123^	52	40	Uncontrolled	Octreotide LAR	AcroQoL, EQ-5D	↑↑ (only AcroQoL)
Pegvisomant weekly or twice weekly	Higham *et al.* (2009) ^128^	7	32	Controlled	Pegvisomant daily	AcroQoL	↔
Pegvisomant - Octreotide LAR	Trainer *et al.* (2009) ^123^	53	40	Uncontrolled	Octreotide LAR	AcroQoL, EQ-5D	↑↑ (only AcroQoL)
Pegvisomant - Octreotide LAR	Neggers *et al.* (2008) ^124^	20	36	Controlled	Octreotide LAR	AcroQoL, PASQ	↑↑
Pegvisomant - Octreotide LAR	Madsen *et al.* (2011) ^125^	18	24	Controlled	Octreotide LAR	EQ-5D, PASQ	↔

↑, improvement in quality of life (QoL) subscale only; ↑↑, improvement in global QoL; ↔, no significant change in QoL; CSKSLPP, Cognitive Scale of Kellner’s Screening List for Psychosocial Problems; EQ-5D, European quality of life scale; KSQ, Kellner’s Symptom Questionnaire (psychological distress and well-being); LAR, long-acting release; NHP, Nottingham Health Profile; PASQ, Patient-Assessed Acromegaly Symptom Questionnaire; SF-36, Short-Form Health Survey; SSS, Signs and Symptoms Scale—acromegaly. This table was adapted from Gadelha MR *et al*., Systemic Complications of Acromegaly and the Impact of the Current Treatment Landscape: An Update, Endocrine Reviews, 2019, Volume 40, Issue 1, Pages 268–332 ^13^ by permission of the Endocrine Society.

Changing long-acting release (LAR) octreotide to pasireotide LAR did not improve QoL even if further disease control was obtained ^122^. On the contrary, in one study, adding pegvisomant to SRLs led to improvement of QoL and clinical symptoms despite not showing any change in circulating IGF-1 ^124, 128^, but studies with a similar design were negative ^125, 129^. This might suggest that improving biochemical control with a favorable safety profile and ease of administration would improve patient QoL. However, further research is required in this area.

Radiotherapy is usually indicated for patients with more aggressive cases of acromegaly, in particular those with residual disease after surgery and medical therapy ^75^. Patients who underwent radiotherapy reported that it had a negative influence on energy, pain, social isolation, physical fatigue, and activity and motivation measured with the MFI-20 ^130^. During follow-up, the scores in 5 out of 26 QoL subscales significantly worsened ^75, 90^. This might be due to a direct brain effect induced by radiotherapy, by new hormonal deficits, by delay in biochemical control of excess GH, or by the disease severity itself or by a combination of these factors. Nonetheless, the results are important to take in account in the decision to irradiate a tumor, especially in young patients.

### Quality of life in acromegaly patients who become growth hormone–deficient after treatment

Development of GH deficiency after treatment of acromegaly has also been shown to affect QoL. Wexler *et al*. found that the mean scores on the Quality of Life Adult Growth Hormone Deficiency Assessment (QoL-AGHDA), SF-36, and Symptom Questionnaire Depression showed significantly impaired QoL in the GH-deficient group when compared with the GH-sufficient group. Peak GH levels after GH-releasing hormone-arginine stimulation were inversely associated with QoL-AGHDA scale scores (R = −0.53; *P* = 0.0005) and Symptom Questionnaire Depression subscale scores (R = −0.35; *P* = 0.031) ^131^.

### Acromegaly-related pain and quality of life

Osteoarthritis is a major cause of pain in acromegaly. Although improvement of hypertrophic articular and periarticular tissues has been noted with normalization of GH/IGF-1, ^132^ degenerative joint disease is somewhat irreversible, and arthralgias might not resolve even after biochemical remission ^133– 135^. Fifty-eight patients with acromegaly were evaluated with the SF-36, Arthritis Impact Measurement Scales 2 (AIMS2), and AcroQoL questionnaires; 52 (90%) reported musculoskeletal pain, 29 (50%) reported neck pain, 49 (84%) had hip osteoarthritis, and 20 (34%) reported knee osteoarthritis. The SF-36, AIMS2, and AcroQoL scores were lower in patients with musculoskeletal pain. The conclusion was that patients with acromegaly had a significantly higher number of musculoskeletal problems that negatively affect their QoL at different levels ^136^. Female patients and those with higher BMI seem to be the most affected ^137^. In regard to vertebral fractures, no definite association has been observed with QoL; this is probably because many events are asymptomatic and many are missed unless patients had vertebral x-ray rather than bone densitometry ^138^.

### Management

QoL is an important treatment objective besides biochemical remission and tumor control. Close attention to patient perception of wellness is essential and should prompt treatment modifications. Use of specific questionnaires helps to identify areas of improvement and monitor therapy effects. Other interventions such as cognitive behavioral therapy with a technique called “Think healthy and feel the difference” have proven sustained improvement over 9 months. ^139^ Moreover, physical activity programs ^140^, and increased knowledge about the disease from physician-guided discussion groups or patient associations may also help to improve patient’s QoL ^141^.

## Conclusions

Recent population studies showed a longer life span for patients with acromegaly; this was most likely due to improvements in treatments, as the average delay in diagnosis seems to be largely unchanged over the decades. Raising awareness of complications of acromegaly even in biochemically controlled disease and introducing adequate screening programs in accordance with Acromegaly Consensus guidelines ^10, 74^ should improve both morbidity and mortality. Patient-reported outcome and QoL have become essential components of monitoring the disease, and improving QoL is an endpoint of the treatment in itself. Although many questionnaires and scales have been developed, more research is needed to determine how best to measure QoL in patients with pituitary disease in general and with acromegaly in particular.
